# Meta-analysis of the effects of exercise intervention on glucose metabolism and body composition in patients with type 2 diabetes mellitus

**DOI:** 10.3389/fendo.2026.1835096

**Published:** 2026-06-29

**Authors:** Yuqing Liu, Mingyuan Zhao, Baoshan Qian

**Affiliations:** 1 Graduate School, Harbin Sport University, Harbin, China; 2 Faculty of Physical Education, Harbin Sport University, Harbin, China

**Keywords:** body composition, exercise intervention, glucose metabolism, meta-analysis, type 2 diabetes mellitus

## Abstract

**Objective:**

To systematically evaluate the effects of exercise intervention on glucose metabolism and body composition in patients with type 2 diabetes mellitus (T2DM).

**Methods:**

Relevant literature built up to May 8, 2026 was searched through Web of Science, PubMed, EMBASE, and Cochrane Library databases, and Meta-analysis was performed using RevMan 5.4 software, and the quality of the literature was evaluated using the Cochrane Risk of Bias Assessment Tool. A total of 16 randomized controlled trials involving 1401 samples were included.

**Results:**

Exercise intervention significantly reduced glycated hemoglobin in patients with T2DM (SMD = -0.46, 95% CI: -0.73 to -0.19, P = 0.0007). Subgroup analysis revealed that combined exercise produced greater improvements (SMD = -0.71, 95% CI: -0.95 to -0.47, P < 0.00001) compared to aerobic exercise alone (SMD = -0.32, 95% CI: -0.62 to 0.02, P = 0.04). Exercise intervention also significantly reduced fasting blood glucose (SMD = -0.52, 95% CI: -0.70 to -0.35, P < 0.00001). However, no statistically significant improvement was observed for the insulin resistance index (SMD = -0.18, 95% CI: -0.40 to 0.04, P = 0.11). Regarding body composition, exercise intervention resulted in a significant reduction in BMI after excluding an outlier study (SMD = -0.37, 95% CI: -0.61 to -0.12, P = 0.003) and significantly improved maximal oxygen uptake (VO_2_max) (SMD = 0.58, 95% CI: 0.34 to 0.82, P < 0.00001).

**Conclusion:**

Exercise intervention can effectively improve glucose metabolism and body composition in patients with T2DM, with mixed (aerobic combined with resistance) exercise showing superior effects on glycemic control compared to aerobic exercise alone. Exercise intervention serves as an important non-pharmacological intervention for the comprehensive management of T2DM.

## 1 Foreword

Type 2 diabetes mellitus (T2DM) has become a major global public health challenge. According to the latest Global Diabetes Map (11th edition) data released by the International Diabetes Federation (IDF) in April 2025, 589 million adults aged 20–79 years with diabetes were already living with diabetes globally in 2024, of whom approximately 252 million were undiagnosed and at higher risk of complications and premature death (1). Furthermore, although glycemic control is a key therapeutic goal for patients with type 2 diabetes, it is cardiovascular disease (CVD), rather than metabolic disorders, that is the primary cause of morbidity and mortality in this patient population (2). These data highlight the centrality of glycemic control in the management of T2DM. As the number of people with type 2 diabetes mellitus continues to rise globally, this trend places a heavy burden on healthcare systems worldwide. This reality underscores the urgent need to comprehensively improve patients cardiovascular health indicators while effectively controlling blood glucose levels.

Exercise intervention, as an important part of the comprehensive management of T2DM, not only improves glycemic control (3) and reduces cardiovascular risk factors, but also aids in weight loss in addition to dietary control and medication, thus enhancing patients’ quality of life. It also helps to prevent the development of type 2 diabetes in high-risk individuals (4). The consensus guidelines of the American Diabetes Association (ADA) and the European Association for the Study of Diabetes (EASD) both list exercise intervention as a first-line non−pharmacological treatment for the management of type 2 diabetes mellitus. Existing studies have shown that the combination of aerobic exercise and resistance training can reduce HbA1c by 0.67%, which corresponds to a 26% reduction in the risk of microvascular complications and a 10% reduction in the incidence of myocardial infarction (5). However, although the overall benefits of exercise intervention have been recognized, a core question remains in clinical practice: do different types of exercise have different effects on glucose metabolism and body composition in patients with type 2 diabetes mellitus? Which exercise regimen provides the greatest metabolic benefits? Answering this question is of great significance for optimizing exercise prescription and achieving individualized diabetes management.

In recent years, a large number of randomized controlled trials have investigated the metabolic effects of exercise interventions in patients with type 2 diabetes mellitus, and the research paradigm has shifted from “whether exercise is effective” to comparative effectiveness studies asking “which type of exercise is more effective.” In terms of glucose metabolism, glycated hemoglobin, as the gold indicator for monitoring glycemic control, is closely related to the development of diabetes-related diseases. Research evidence shows that for every 1% increase in HbA1c level, the risk of CVD increases by 1.18% (6); whereas for every 1% decrease in HbA1c, the risk of microvascular complications can be reduced by 37% and the risk of myocardial infarction by 14% (7). This fully reflects the important value of HbA1c in assessing the condition of type 2 diabetes mellitus patients and preventing the aftermath. Fasting blood glucose, in combination with glycated hemoglobin, is widely used to assess the adequacy of glycemic control in diabetes care. Its normalization is considered a reliable indicator of good metabolic control in type 2 diabetes (8). Several studies have shown a significant association between fasting glucose and the development of diabetic retinopathy, including Pima Indians (9) and Egyptians (10). In addition, insulin resistance is a distinguishing feature of type 2 diabetes (11), which is present and predicts the onset of the disease years before it occurs (12). It is associated not only with the metabolism of blood glucose, but also with the development of several complications such as cardiovascular disease.

In terms of body composition, the association between BMI and type 2 diabetes has been widely recognized (13–17). Since weight loss in diabetic patients improves glycemic control in the short term (18), weight loss is essential for the treatment of type 2 diabetes. Maximum oxygen uptake, which is the gold index for assessing cardiovascular fitness and exercise capacity, is also important in patients with type 2 diabetes. Research evidence indicates that maximal oxygen uptake is significantly lower in patients with type 2 diabetes mellitus compared with healthy individuals, with a reduction of approximately 15-20%. This difference may be attributable to pathophysiological mechanisms such as hyperglycemia-induced endothelial dysfunction, reduced mitochondrial density, and decreased efficiency of oxygen delivery and utilization (19). Lemura et al. conducted a meta-analysis of maximal oxygen uptake in older adults and found that the majority of the studies demonstrated significant elevations in maximal oxygen uptake. Despite the inevitable decline in maximal oxygen uptake with age, aerobic exercise training can still promote favorable adaptations in maximal oxygen uptake in older adults (20). Despite the large number of existing studies, several key gaps remain in the current evidence. First, there is a lack of systematic direct comparisons between different exercise types, and the evidence directly comparing combined training with aerobic exercise alone is insufficient. Second, substantial variations across studies in exercise intensity, frequency, intervention duration, and baseline participant characteristics have led to considerable heterogeneity in meta−analysis results. Third, existing meta−analyses have mostly focused on HbA1c, with limited comprehensive evaluation of insulin resistance, body composition, and cardiorespiratory fitness.

Based on the above background, this systematic review and meta-analysis was conducted to comprehensively evaluate the effects of exercise interventions on glucose metabolism and body composition in patients with type 2 diabetes mellitus. The present study adopted the following core design: glucose metabolism indicators (HbA1c, fasting blood glucose, and the homeostatic model assessment for insulin resistance) together with body composition and cardiorespiratory fitness indicators (body mass index and maximal oxygen uptake) were included to comprehensively assess the multi-dimensional metabolic benefits of exercise interventions. Following the recommendations of the Cochrane Handbook, the online tool StatsToDo was used to combine the two exercise intervention arms in the same study into a single “combined exercise intervention group” before comparison with the control group, thereby effectively avoiding unit−of−analysis errors. Subgroup analyses (stratified by exercise type: aerobic exercise vs. combined exercise) and sensitivity analyses (leave−one−out approach) were performed to systematically identify sources of heterogeneity. The GRADE approach was used to assess the quality of evidence for each outcome. This study aimed to answer the following core questions: (1) Can exercise intervention significantly improve glucose metabolism and body composition indicators in patients with type 2 diabetes mellitus? (2) Is combined exercise superior to aerobic exercise alone? (3) Which factors are the main sources of heterogeneity? The findings will provide evidence−based support for optimizing exercise prescriptions in patients with type 2 diabetes mellitus and contribute to the development of precise non−pharmacological treatment strategies.

## 2 Research methods

### 2.1 Literature search

This systematic review is reported in accordance with the PRISMA 2020 guidelines and strictly adheres to the following PICOS framework: Population: Patients with a standard diagnosis of type 2 diabetes mellitus (aged ≥18 years), with no restrictions on disease duration or medication regimen. Intervention: Any form of structured exercise intervention, including aerobic exercise, resistance training, combined training, and high-intensity interval training. Comparator: Usual care, no exercise intervention, or a non-exercise control group. Outcomes: The primary outcome measures are glycated hemoglobin and fasting blood glucose; the secondary outcome measures are maximal oxygen uptake, body mass index, and the homeostatic model assessment for insulin resistance. Study design: Randomized controlled trials.

Relevant studies was searched in Web of Science, EBSCO, PubMed, Embase, and The Cochrane Library databases from the inception of the database to May 8, 2026. English search terms: Young Adult, Diabetes Mellitus, Type2, T2DM, Exercise, Physical Exercise, Aerobic Exercise, Isometric Exercise, Acute Exercise, Exercise Training, Physical Activity and Randomized Controlled Trial, etc., while tracing the references of related literature to supplement the literature. Taking Web of Science and Cochrane Library as an example, the literature search strategy is shown in **Table 1**. All search strategies are provided in the **Supplementary Material**.

**Table 1:** Web of Science literature search strategy.

Database	Search strategy	Number
Web Of Science	((((((((((((((((((((((((TS=(Exercise)) OR TS=(Exercises)) OR TS=(Exercise, Physical)) OR TS=(Exercises, Physical)) OR TS=(Physical Exercise)) OR TS=(Physical Exercises)) OR TS=(Exercise, Aerobic)) OR TS=(Aerobic Exercise)) OR TS=(Aerobic Exercises)) OR TS=(Exercises, Aerobic)) OR TS=(Exercise, Isometric)) OR TS=(Exercises, Isometric)) OR TS=(Isometric Exercises)) OR TS=(Isometric Exercise)) OR TS=(Acute Exercise)) OR TS=(Acute Exercises)) OR TS=(Exercise, Acute)) OR TS=(Exercises, Acute)) OR TS=(Exercise Training)) OR TS=(Exercise Trainings)) OR TS=(Training, Exercise)) OR TS=(Trainings, Exercise)) OR TS=(Physical Activity)) OR TS=(Activities, Physical)) OR TS=(Activity, Physical)) OR TS=(Physical Activities)) AND (((TS=(Young Adult)) OR TS=(Adults, Young)) OR TS=(Adult, Young) OR TS=(Young Adults))) AND (((TS=(Diabetes Mellitus, Type 2)) OR TS=(Diabetes Mellitus, Type II)) OR TS=(Type 2 Diabetes Mellitus) OR TS=(T2DM))) AND TS=(Randomized Controlled Trial)	395
Cochrane Library	MeSH descriptor: [Young Adult] explode all trees OR (Adults, Young):ti,ab,kw or (Adult, Young):ti,ab,kw or (Young Adults):ti,ab,kw AND MeSH descriptor: [Diabetes Mellitus, Type 2] explode all trees OR (Diabetes Mellitus, Type II):ti,ab,kw or (Type 2 Diabetes Mellitus):ti,ab,kw or (T2DM):ti,ab,kw AND MeSH descriptor: [Exercise] explode all trees OR (Exercises):ti,ab,kw or (Exercise, Physical):ti,ab,kw or (Exercises, Physical):ti,ab,kw or (Physical Exercise):ti,ab,kw or (Physical Exercises):ti,ab,kw or (Exercise, Aerobic):ti,ab,kw or (Aerobic Exercise):ti,ab,kw or (Aerobic Exercises):ti,ab,kw or (Exercises, Aerobic):ti,ab,kw or (Exercise, Isometric):ti,ab,kw or (Exercises, Isometric):ti,ab,kw or (Isometric Exercises):ti,ab,kw or (Isometric Exercise):ti,ab,kw or (Acute Exercise):ti,ab,kw or (Acute Exercises):ti,ab,kw or (Exercise, Acute):ti,ab,kw or (Exercises, Acute):ti,ab,kw or (Exercise Training):ti,ab,kw or (Exercise Trainings):ti,ab,kw or (Training, Exercise):ti,ab,kw or (Trainings, Exercise):ti,ab,kw or (Physical Activity):ti,ab,kw or (Activities, Physical):ti,ab,kw or (Activity, Physical):ti,ab,kw or (Physical Activities):ti,ab,kw AND (Randomized Controlled Trial):ti,ab,kw	254

### 2.2 Literature inclusion and exclusion criteria

Inclusion criteria: (1) it must be related to both exercise intervention and type 2 diabetes; (2) the type of study is a randomized controlled trial (RCT) and the included literature is full text, which must be in English; (3) the literature’s outcome metrics must be in line with the purpose of the study; and (4) the study population is adult (≥18 years old) patients.

Exclusion criteria: (1) Duplicate literature; (2) Literature with unavailable data; (3) Literature that does not meet the inclusion criteria.

### 2.3 Literature screening and data extraction

In this systematic review, each study was independently assessed by two reviewers (LYQ and ZMY) based on predetermined eligibility criteria. Any disagreements were resolved through discussion or by consultation with a third reviewer (QBS). When screening the literature, we first read the title and abstract, and then read the full text after excluding obviously irrelevant literature to determine whether it would be included in the final study. Data were extracted using a standardized form, which included: first author, year of publication, country, subject characteristics (sample size, age, BMI), and intervention characteristics (type of intervention, periodicity, frequency).

### 2.4 Literature quality evaluation

Literature quality evaluation was performed according to the risk of bias assessment tool version 2.0 (RoB 2.0) (21) recommended by the Cochrane Systematic Evaluator’s Manual 6.1. Two researchers evaluated the included literature for possible bias in each of the five areas of the randomization process, deviation from the established interventions, missing data for the endpoints, the process of measurement of the endpoints, and the reporting of the outcomes, and summarized the results of the risk of cheapness assessment. In case of disagreement during the evaluation process, the decision was discussed and negotiated.

Two authors (LYQ and ZMY) evaluated the quality of evidence using the Grading of Recommendations Assessment, Development and Evaluation (GRADE) framework (22), assessing study design, risk of bias, heterogeneity, indirectness (PICO), imprecision (event counts, confidence intervals, and sample size), and publication bias. Each criterion was rated as “not serious,” “serious,” or “very serious”; a rating of “serious” or “very serious” led to a downgrade of one or two levels, respectively. The overall quality of the evidence was then classified into four levels: high, moderate, low, or very low.

### 2.5 Statistical methods

For this study, the mean and standard deviation (Mean ± SD) at baseline and post-intervention were extracted from the original studies, and the change from baseline was calculated for the meta-analysis. All calculated change values have been uploaded as **Supplementary Material** (**Supplementary Table 1**).The extracted data were tested for heterogeneity, were merged, subgroup analyses were performed, and forest plots were generated using RevMan 5.4 software. Standardized mean difference (SMD) was used as the effect size for all outcomes to maintain methodological consistency. In addition, to facilitate clinical interpretation, mean difference (MD) was also calculated for outcomes measured on comparable scales (HbA1c and BMI), and the results are provided as **Supplementary Material** (**Supplementary Figure 1**; **Supplementary Figure 2**). SMD effect size was evaluated as follows: 0.2 ≤ SMD < 0.5 as a small effect size, 0.5 ≤ SMD < 0.8 as a medium effect size, and SMD ≥ 0.8 as a large effect size (23).

Heterogeneity between studies was tested using the I² statistic. When P ≥ 0.1 and I² < 25%, it was considered that there was no significant heterogeneity among studies; when 25% < I² < 50%, it was considered that there was moderate heterogeneity among studies. A fixed-effects model was used for analysis when heterogeneity was low. When P < 0.1 and I² > 75%, it was considered that there was substantial heterogeneity among studies (24). When the heterogeneity was large, a random-effects model was used to combine the effect sizes (25). This was because the random-effects model had a wider confidence interval compared to the fixed-effects model.

For randomized controlled trials that included two exercise intervention arms sharing a single control group, to avoid a unit-of-analysis error, we followed the recommendations of the Cochrane Handbook and combined the two exercise intervention arms into a single “combined exercise intervention group”. The combination was performed using the online tool StatsToDo (Combining n, mean and SD from multiple groups, URL: https://www.statstodo.com/CombineMeansSDs.php). In this study, all three-arm trials that met this criterion (26–29) were combined using this method. Subsequently, the resulting “combined exercise intervention group” was compared with the shared control group, so that each study contributed only one independent effect size to the meta-analysis, effectively avoiding the pseudo-replication problem caused by repeated use of the control group sample size.

## 3 Results

### 3.1 Results of literature search and screening

**Figure 1:**
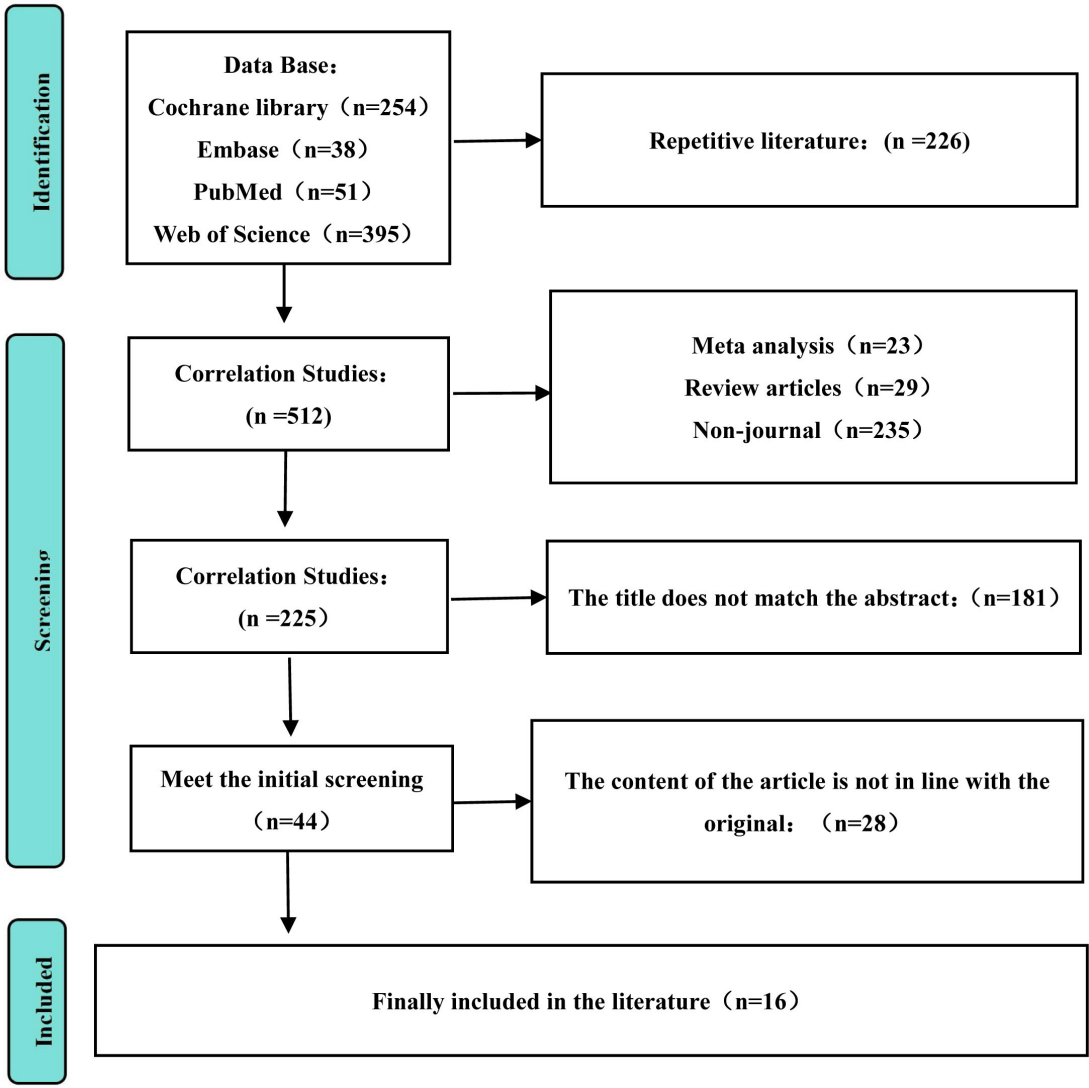
Overall bias risk assessment for inclusion studies.

A total of 738 articles were retrieved from English databases, all of which were in English, 225 articles entered into the reading of the title and abstract screening, 44 articles entered into the full-text reading of the screening, and the full-text reading excluded the literature that the outcome indicators did not meet and the inability to extract the amount of the effect, and ultimately included in the literature of 16 articles, and the process of the screening of the literature is shown in **Figure 1**.

### 3.2 Basic characteristics and quality evaluation of the included literature

**Figure 2:**
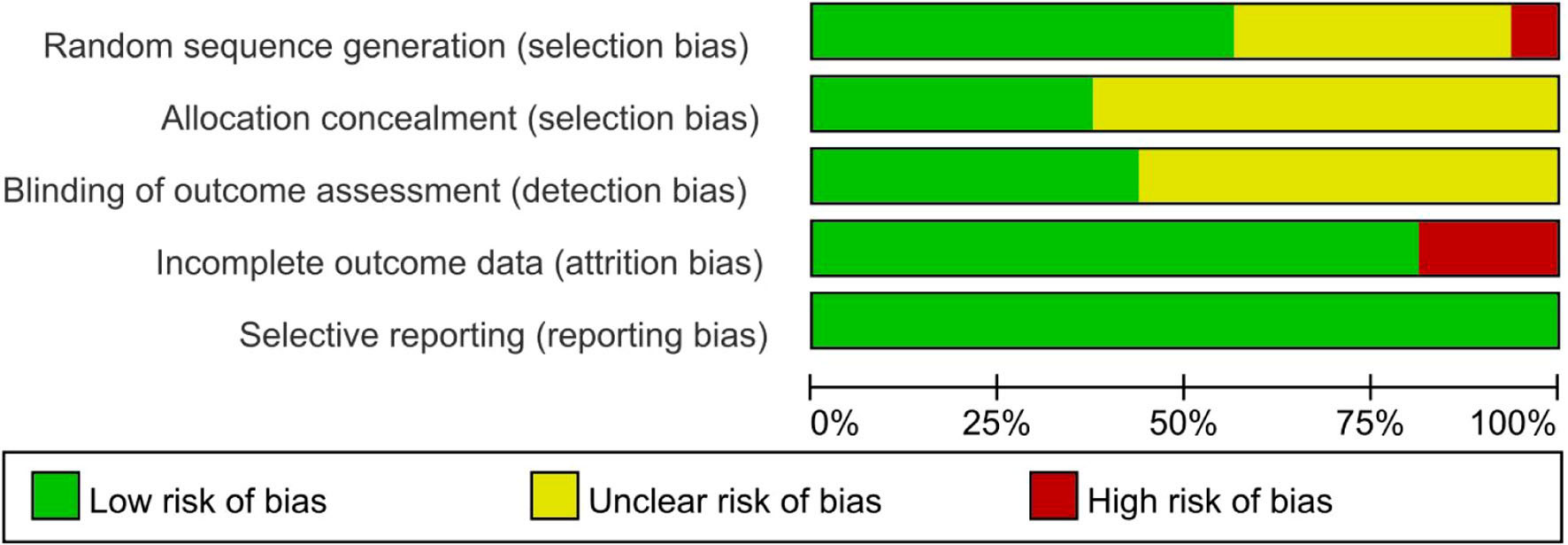
Flow chart of literature screening.

**Figure 3:**
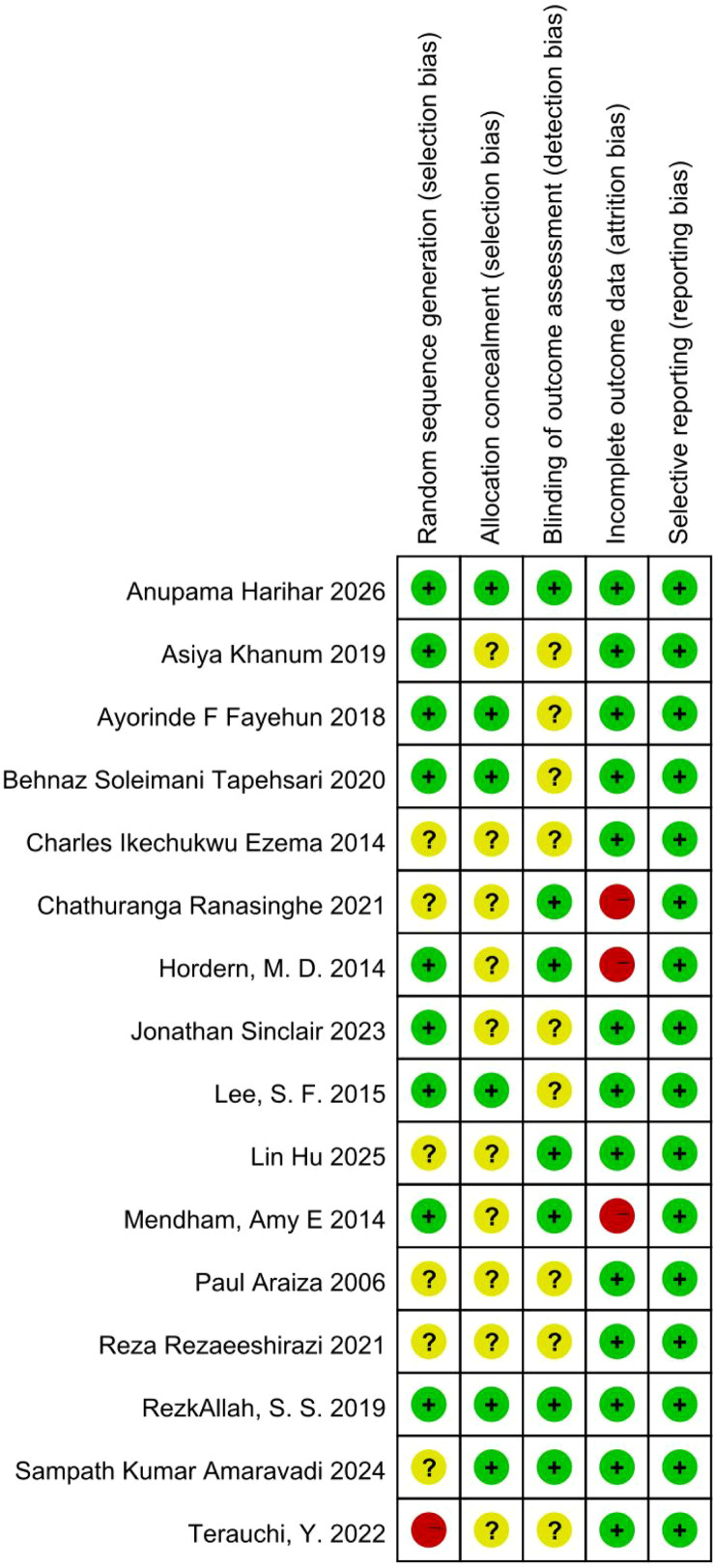
Individual bias risk assessment diagram included in the study.

Sixteen literature were RCTs, of which two was evaluated as low risk of bias, ten as some concerns, and four as high risk of bias according to Cochrane Risk of Bias Assessment Criteria, as shown in **Figures 2**, **3** and **Tables 2**, **3**.

**Table 2:** Subject characteristics of the literature.

Characteristics of the literature		Subject characteristics					
Author and year	Country	Experimental group			Control group		
		Sample capacity	Age	BMI	Sample capacity	Age	BMI
Terauchi 2022 (30)	Japan	n=110(42F/68M)	54.6 ± 9.2	26.66 ± 3.77	n=114(45F/69M)	55.7 ± 10.2	26.25 ± 3.59
Hordern 2009 (31)	Australia	n=88(42F/46M)	56.1 ± 11.7	/	n=88(30F/50M)	55 ± 8.5	/
Fayehun 2018 (32)	Nigeria	n=23(15F/8M)	53.96 ± 7.7	21.73 ± 3.38	n=23(14F/9M)	53.96 ± 7.7	23.08 ± 3.26
Mendham 2015 (33)	Australia	n=11	39.5 ± 10.6	31.6 ± 3.1	n=10	36.1 ± 16.1	34.5 ± 6.6
Hu 2025 (34)	China	n=50(22F/28M)	65.50 ± 4.5	25.17 ± 3.30	n=50(23F/27M)	66.52 ± 5.0	24.3 ± 3.68
Ezema 2014 (35)	Nigeria	n=30	47.53 ± 4.68	22.60 ± 0.93	n=24	47.13 ± 4.48	23.06 ± 0.86
Araiza 2006 (36)	USA	n=15	49 ± 11	30.0 ± 4.4	n=15	51 ± 10	33.5 ± 6.6
Amaravadi 2024 (37)	UK	n=75(17F/58M)	56.05 ± 8.77	25.32 ± 3.16	n=71(32F/39M)	53.90 ± 10.2	26.27 ± 3.63
Lee 2015 (26)	China	n=80(40F/40M)	55.28 ± 8.98	25.72 ± 3.65	n=40(22F/18M)	56.07 ± 9.41	26.78 ± 3.50
RezkAllah 2019 (27)	Egypt	n=40(19F/21M)	31.4 ± 5.23	28.22 ± 1.39	n=20(8F/12M)	35.9 ± 5.89	28.41 ± 1.37
Rezaeeshirazi 2021 (28)	Iran	n=27	21.18 ± 2.12	32.33 ± 1.52	n=15	22.47 ± 2.03	32.16 ± 1.50
Sinclair 2023 (38)	UK	n=27(14F/13M)	42.07 ± 9.72	29.44 ± 4.38	n=26(12F/14M)	45.88 ± 8.51	29.73 ± 6.24
Tapehsari 2020 (39)	Iran	n=47(38F/9M)	45.85 ± 6.85	27.92 ± 3.48	n=48(41F/7M)	46.58 ± 5.31	29.24 ± 4.10
Khanum 2019 (40)	Pakistan	n=32	43.28 ± 2.95	/	n=32	44.34 ± 3.76	/
Ranasinghe 2021 (29)	Sri Lanka	n=56(32F/24M)	50.5 ± 9.54	/	n=30(14F/16M)	49.3 ± 7.0	/
Harihar 2026 (41)	India	n=42	34 ± 5.14	28.4 ± 4.56	n=42	34.5 ± 4.09	29.9 ± 4.85

Values are expressed as mean ± standard deviation (SD) or number of participants (percentage). F, female; M, male; BMI, body mass index. “/” indicates that the data were not available or not reported in the original study.

**Table 3:** Intervention characteristics of the literature.

Characteristics of the literature		Intervention features				
Author and year	Country	Experimental group			Control group	
		Type of intervention	Period	Frequency	Type of intervention	Period
Terauchi 2022 (30)	Japan	Aerobic Exercise+Resistance Exercise	13weeks	3days/week	Dietary therapy and physical activity in daily life	13weeks
Hordern 2009 (31)	Australia	Aerobic Exercise+Resistance Exercise	48weeks	150min/week	Usual Care	48weeks
Fayehun 2018 (32)	Nigeria	10,000 Steps	10weeks	7days/week	Normal living habits	10weeks
Mendham 2015 (33)	Australia	Strength Exercise, Core Exercise, Aerobic Exercise	12weeks	3days/week	Normal living habits	12weeks
Hu 2025 (34)	China	Home Resistance Band Exercise	12weeks	3days/week	Usual Care	12weeks
Ezema 2014 (35)	Nigeria	Continuous Exercise (Aerobic Exercise)	8weeks	3days/week	Normal living habits	8weeks
Araiza 2006 (36)	USA	10,000 Steps	6weeks	7days/week	Normal living habits	6weeks
Amaravadi 2024 (37)	UK	Aerobic Exercise+Resistance Exercise	12weeks	3-5days/week	Usual Care	12weeks
Lee 2015 (26)	China	AEG Aerobic Exercise Group+AMSG Accumulates Millions of Steps	12weeks	5days/week	CG Usual Care	12weeks
RezkAllah 2019 (27)	Egypt	LV-HIT+HV-HIT	12weeks	3days/week	Normal living habits	12weeks
Rezaeeshirazi 2021 (28)	Iran	Aerobic Exercise+Resistance Exercise	8weeks	4days/week	Control Group	8weeks
Sinclair 2023 (38)	UK	Home-based Physical Activity	12weeks	5days/week	Usual Care	12weeks
Tapehsari 2020 (39)	Iran	PAP+Lifestyle Education	12weeks	5days/week	Lifestyle education	12weeks
Khanum 2019 (40)	Pakistan	Diaphragmatic Breathing Exercises	12weeks	/	Usual Care	12weeks
Ranasinghe 2021 (29)	Sri Lanka	Aerobic Exercise+Resistance Exercise	12weeks	2days/week	Usual Care	12weeks
Harihar 2026 (41)	India	Aerobic Exercise+Resistance Exercise	24weeks	3-5days/week	Usual Care	24weeks

### 3.3 Meta-analysis of the effects of exercise intervention on patients with type 2 diabetes mellitus

#### 3.3.1 Meta-analysis of the effect size of glycated hemoglobin

**Figure 4:**
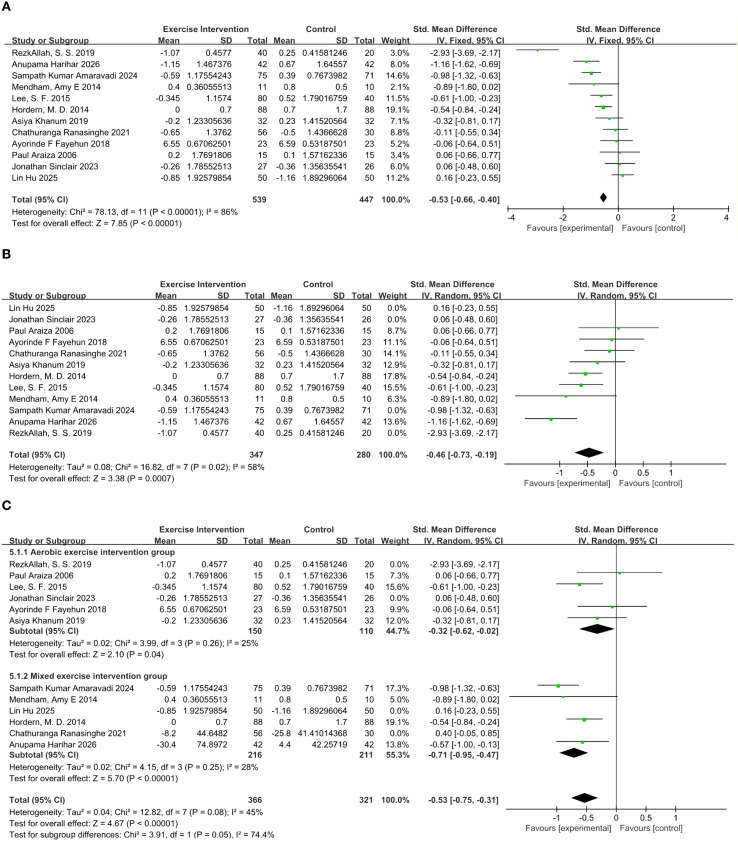
Forest plot showing the effect of exercise intervention on glycated hemoglobin (HbA1c) in patients with type 2 diabetes mellitus: **(A)** overall analysis including all 12 studies; **(B)** sensitivity analysis after excluding four outlier studies; **(C)** subgroup analysis stratified by exercise type.

A total of 12 studies were included in the analysis, three of which (26, 27, and 29) were three-arm randomized controlled trials (containing two different types of exercise intervention groups and one control group). To avoid a unit-of-analysis error, the two exercise intervention arms in these three studies were combined into a single combined exercise intervention group before being compared with the control group, so that each study contributed only one independent effect size. The initial meta-analysis showed substantial heterogeneity (I² = 86%, P < 0.00001) (**Figure 4A**). A leave-one-out sensitivity analysis revealed that after excluding four studies (27, 34, 37, 38), the heterogeneity was significantly reduced. Therefore, the primary analysis was based on the remaining eight studies after excluding these four outliers. The primary analysis showed that exercise intervention significantly reduced HbA1c levels in patients with type 2 diabetes mellitus, with a pooled effect size of SMD = -0.46 (95% CI: -0.73 to -0.19, P = 0.0007) and heterogeneity of I² = 58% (**Figure 4B**). Subgroup analyses stratified by exercise type (**Figure 4C**) showed that combined exercise significantly reduced HbA1c (SMD = -0.71, 95% CI: -0.95 to -0.47, P < 0.00001, I² = 28%), and aerobic exercise also significantly reduced HbA1c (SMD = -0.32, 95% CI: -0.62 to -0.02, P = 0.04, I² = 25%). The test for subgroup differences showed a significant difference between the two groups (P = 0.05), indicating that combined exercise was significantly superior to aerobic exercise in reducing HbA1c.

To further enhance clinical interpretability, we also performed sensitivity analysis using mean difference (MD) for HbA1c. A leave-one-out sensitivity analysis revealed that after excluding four studies (27, 34, 37, 41), heterogeneity decreased from 85% to 37%. The pooled MD was -0.36 (95% CI: -0.60 to -0.12, P = 0.003), corresponding to an average absolute reduction of 0.36% in HbA1c (**Supplementary Figure 1**). Although the excluded studies differed slightly between the SMD and MD sensitivity analyses, both approaches consistently confirmed that exercise intervention significantly reduced HbA1c levels.

#### 3.3.2 Meta-analysis of the effect size of fasting blood glucose

**Figure 5:**
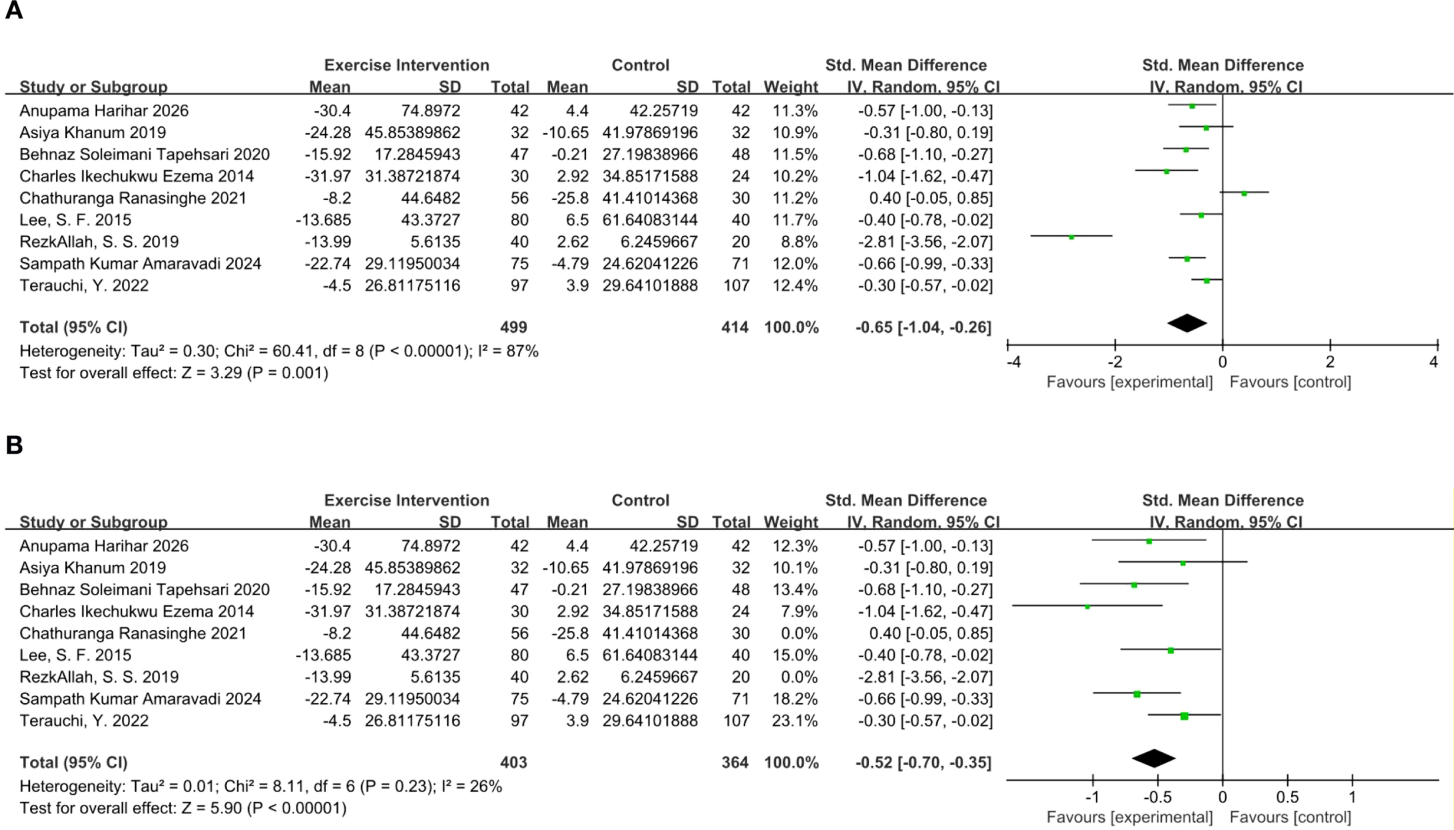
Forest plot showing the effect of exercise intervention on fasting blood glucose (FBG) in patients with type 2 diabetes mellitus: **(A)** Overall analysis (9 studies); **(B)** Sensitivity analysis (excluding 2 outlier study).

A total of nine studies were included in the analysis, three of which (26, 27, 29) were three-arm randomized controlled trials. As with the HbA1c analysis, the two exercise intervention arms in these three studies were combined into a single combined exercise intervention group before being included in the analysis. The initial meta-analysis showed substantial heterogeneity (I² = 87%, P < 0.00001) (**Figure 5A**). A leave-one-out sensitivity analysis revealed that after excluding two studies (27, 29), the heterogeneity was reduced to an acceptable level (I² = 26%, P = 0.23), indicating that these two studies were the main sources of heterogeneity. The meta-analysis (based on seven studies after excluding the two outliers) showed that exercise intervention significantly reduced fasting blood glucose levels in patients with type 2 diabetes mellitus, with a pooled effect size of SMD = -0.52 (95% CI: -0.70 to -0.35, P < 0.00001) and heterogeneity of I² = 26% (**Figure 5B**).

#### 3.3.3 Meta-analysis of the effect size of the homeostatic model assessment for insulin resistance

**Figure 6:**
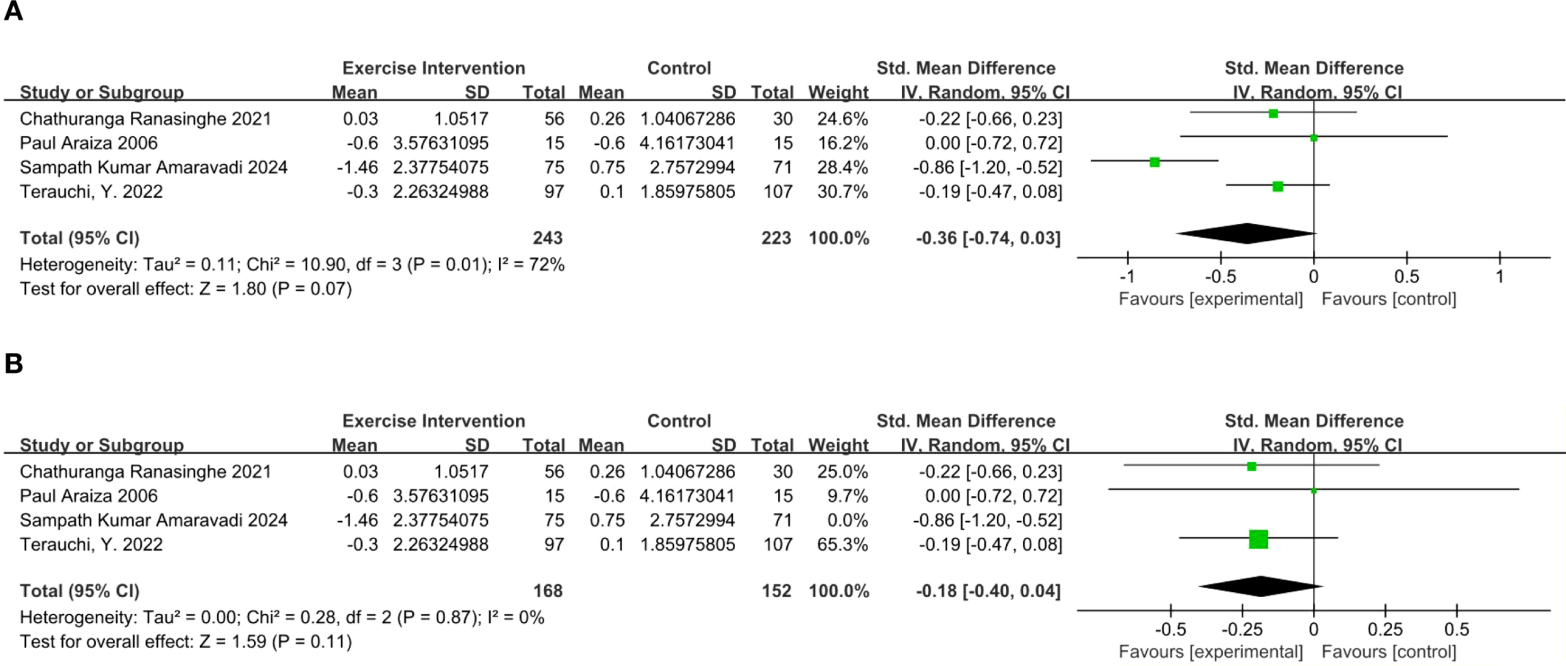
Forest plot of HOMA-IR for exercise intervention in type 2 diabetes mellitus. **(A)** Overall analysis (4 studies); **(B)** Sensitivity analysis (excluding 1 outlier study).

A total of four studies were included in the analysis, one of which (29) was a three-arm randomized controlled trial. To avoid a unit-of-analysis error, the two exercise intervention arms in this three-arm trial were combined into a single combined exercise intervention group before being included in the analysis. The initial meta-analysis showed a trend toward a reduction in HOMA-IR following exercise intervention, but it did not reach statistical significance, with a pooled effect size of SMD = -0.36 (95% CI: -0.74 to 0.03, P = 0.07) and heterogeneity of I² = 72% (**Figure 6A**). A leave-one-out sensitivity analysis revealed that after excluding the study by Sampath Kumar Amaravadi (37), the heterogeneity was reduced to a perfect level (I² = 0%, P = 0.87), indicating that this study was the main source of heterogeneity. This study reported a substantially larger effect size (SMD = -0.86) than the other studies. The sensitivity analysis (based on three studies after excluding Sampath Kumar 2024) showed a pooled effect size of SMD = -0.18 (95% CI: -0.40 to 0.04, P = 0.11) and heterogeneity was reduced to I² = 0% (**Figure 6B**).

#### 3.3.4 Meta-analysis of the effect size of body mass index

**Figure 7:**
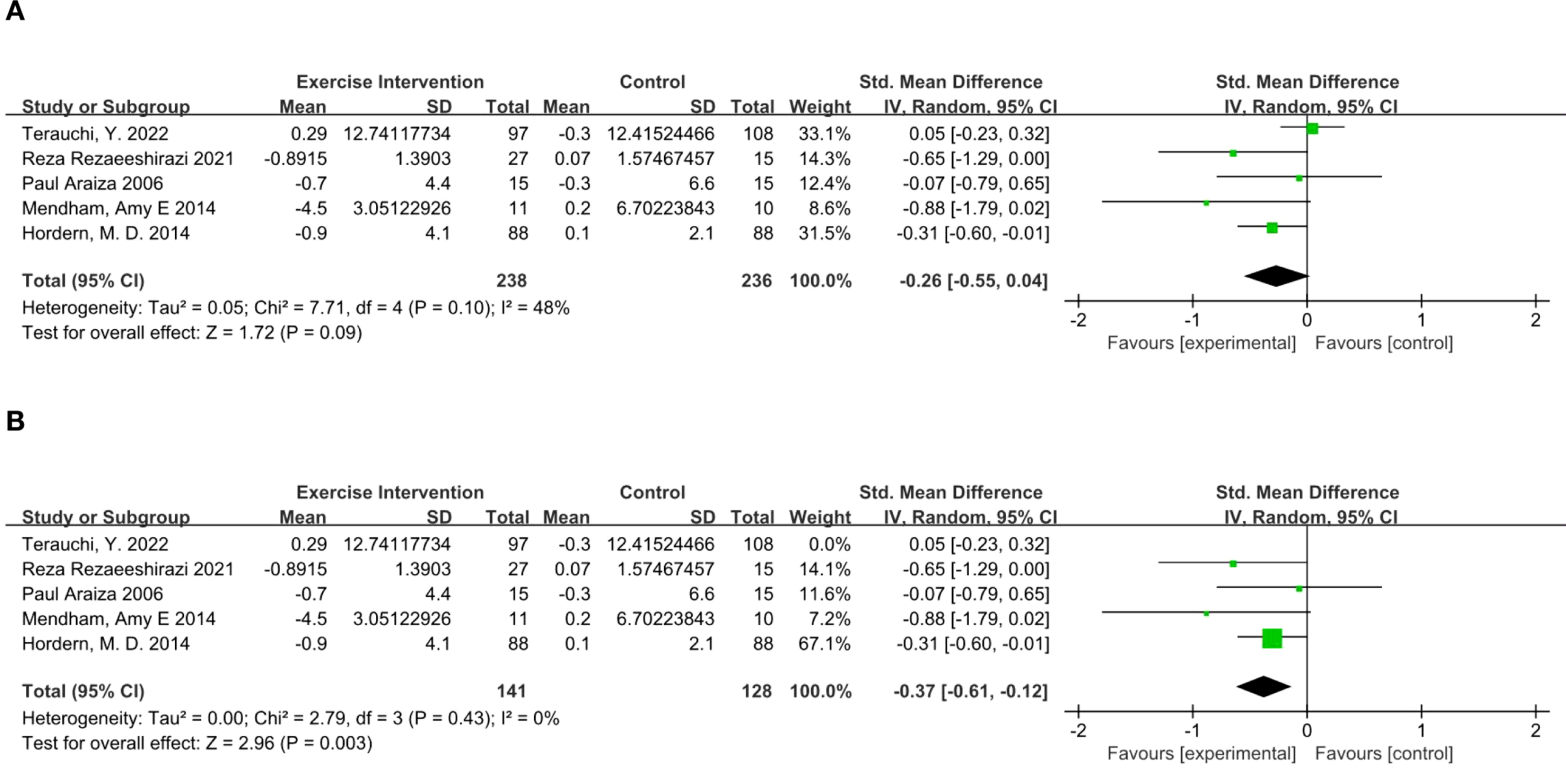
Forest plot showing the effect of exercise intervention on body mass index (BMI) in patients with type 2 diabetes mellitus: **(A)** Overall analysis (5 studies); **(B)** Sensitivity analysis (excluding 1 outlier study).

A total of five studies were included in the analysis, one of which (28) was a three-arm randomized controlled trial. To avoid a unit-of-analysis error, the two exercise intervention arms in this three-arm trial were combined into a single combined exercise intervention group before being included in the analysis. The initial meta-analysis showed a pooled effect size of SMD = -0.26 (95% CI: -0.55 to 0.04, P = 0.09) with heterogeneity of I² = 48% (**Figure 7A**). A leave-one-out sensitivity analysis revealed that the study by Terauchi (30) had an effect direction (SMD = +0.05) opposite to that of the other four studies and carried the highest weight (33.1%), making it the main source of heterogeneity. This study reported a slight increase in BMI following exercise intervention, which lacks clear biological plausibility. Therefore, after excluding Terauchi (30), the results based on four studies showed that exercise intervention significantly reduced BMI in patients with type 2 diabetes mellitus, with a pooled effect size of SMD = -0.37 (95% CI: -0.61 to -0.12, P = 0.003) and heterogeneity of I² = 0% (**Figure 7B**).To facilitate clinical interpretation, mean difference (MD) analysis was also performed. The MD results showed that exercise intervention reduced BMI by an average of 0.99 kg/m² (95% CI: -1.64 to -0.34, P = 0.003), with heterogeneity of I² = 0% (**Supplementary Figure 2**).

#### 3.3.5 Meta-analysis of the effect size of maximal oxygen uptake

**Figure 8:**
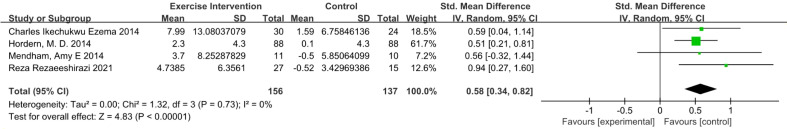
Forest plot of the effect of exercise intervention on VO_2_max in patients with type 2 diabetes mellitus.

A total of four studies were included in the analysis, one of which (28) was a three-arm randomized controlled trial. To avoid a unit-of-analysis error, the two exercise intervention arms in this three-arm trial were combined into a single combined exercise intervention group before being included in the analysis. A random-effects model was used for the meta-analysis. The heterogeneity test showed no statistical heterogeneity among the studies (I² = 0%, P = 0.73). The results indicated that exercise intervention significantly improved VO_2_max levels in patients with type 2 diabetes mellitus, with a pooled effect size of SMD = 0.58 (95% CI: 0.34 to 0.82, Z = 4.83, P < 0.00001) (**Figure 8**). The forest plot showed that the effect sizes of all four studies were in the same direction (all positive). The study by Hordern 2009 (31) received the highest weight (61.7%) due to its largest sample size, with an effect size of SMD = 0.51 (95% CI: 0.21 to 0.81).

## 4 Discussion

### 4.1 Effects of exercise intervention on outcome measures

#### 4.1.1 Glycated hemoglobin

This meta-analysis found that exercise intervention significantly reduced HbA1c levels in patients with type 2 diabetes mellitus (SMD = -0.46, 95% CI: -0.73 to -0.19, P = 0.0007). Subgroup analysis further revealed that combined exercise was significantly more effective (SMD = -0.71) than aerobic exercise alone (SMD = -0.32), with a statistically significant difference between subgroups (P = 0.05). This finding is consistent with previous meta-analyses (42, 43), suggesting that combined exercise should be prioritized in diabetes management. The initial analysis showed substantial heterogeneity (I² = 86%). Through leave-one-out sensitivity analysis, we identified four outlier studies. Among these, the study by RezkAllah et al. (27) showed an extremely large effect size (SMD = -2.93). This study enrolled prediabetic individuals (HbA1c 5.7–6.4%) with preserved β−cell function, making them more responsive to exercise (44, 45); the participants were young adults (mean age 31.8 years), whose mitochondrial oxidative capacity and skeletal muscle plasticity are superior to those of older populations (46); the 90% HRmax high-intensity interval training produced greater improvements in glycemic control than moderate-intensity continuous training (47, 48); and the concomitant low-calorie diet (1,200–1,800 kcal/day) further amplified the glucose-lowering effect (49). In contrast, the studies by Lin Hu et al. (34) and Sinclair et al. (38) showed positive effect directions (SMD = +0.16 and +0.06, respectively). The former enrolled pre-frail older adults (mean age 66 years); frailty is associated with sarcopenia and impaired mitochondrial function, which may blunt the glycemic response to exercise (50, 51). Moreover, the low-intensity resistance training (50–65% HRmax) used in that study may be insufficient to stimulate glucose uptake in insulin-resistant skeletal muscle (52). The latter was conducted in Saudi Arabia, where cultural barriers significantly limit physical activity among women (53), and the unsupervised home-based exercise model generally results in low adherence (54). The study by Sampath Kumar et al. (UK) showed a relatively large effect size (SMD = -0.98), attributable to its high-frequency (3–5 sessions/week) high-intensity combined exercise protocol, which produced a synergistic effect of aerobic and resistance training (55, 56). From a clinical perspective, based on mean difference analysis (**Supplementary Figure 1**), exercise intervention reduced HbA1c by approximately 0.36% (95% CI: -0.60% to -0.12%). According to the UK Prospective Diabetes Study (UKPDS), each 1% reduction in HbA1c is associated with a 37% reduction in microvascular complications and a 14% reduction in myocardial infarction risk (57). Although a 0.36% reduction is smaller than that achieved with pharmacotherapy, exercise as an adjunctive non-pharmacological intervention may have additive effects when combined with conventional glucose-lowering medications. For patients with baseline HbA1c > 8.5%, the benefits of exercise may be more pronounced (58). In summary, combined aerobic and resistance exercise should be prioritized, although individualized parameters of exercise prescription require further investigation.

#### 4.1.2 Fasting blood glucose

Exercise intervention also significantly reduced fasting blood glucose levels in patients with type 2 diabetes mellitus (SMD = -0.52, 95% CI: -0.70 to -0.35, P < 0.0001), with heterogeneity reduced to an acceptable level after sensitivity analysis (I² = 26%). The heterogeneity in the initial analysis originated mainly from two studies. The study by Ranasinghe et al. showed an effect direction opposite to that of other studies (SMD = +0.40), a finding that warrants attention. In that study, the control group experienced an abnormal reduction in fasting blood glucose of 25.8 mg/dL (16.4%), while the resistance exercise group showed a slight increase. This phenomenon may be related to the unique “thin-fat” phenotype of South Asian populations—characterized by high body fat percentage and insulin resistance despite normal BMI—whose metabolic response to exercise may differ from that of Caucasian populations (59). Furthermore, the exercise frequency was only 2 days per week, lower than the 3–5 days per week recommended in current guidelines (60), and the regular contact with the control group may have induced a Hawthorne effect. The study by RezkAllah et al. (27) showed an extremely large effect size (SMD = -2.81), the mechanism of which is consistent with that described for HbA1c: the additive effects of a prediabetic population, high-intensity interval training, and a low-calorie diet. High-intensity interval training acutely increases skeletal muscle glucose uptake through both insulin-dependent and insulin-independent pathways, primarily via AMPK activation, which enhances GLUT4 translocation to the cell membrane (61, 62). Elevated fasting blood glucose stimulates hepatic glucose output, creating a vicious cycle of hyperglycemia (63). The observed reduction in fasting blood glucose of approximately 0.52 SMD units has clear clinical significance and is consistent with previous studies reporting that structured physical activities such as yoga improve fasting blood glucose (64).

#### 4.1.3 Homeostatic model assessment for insulin resistance.

Exercise intervention did not achieve statistical significance for improving HOMA-IR (SMD = -0.36, 95% CI: -0.74 to 0.03, P = 0.07), and substantial heterogeneity was present (I² = 72%). After excluding the study by Sampath Kumar et al. (UK), heterogeneity completely disappeared (I² = 0%), but the pooled effect size decreased to a non-significant level (SMD = -0.18, P = 0.11). The effect size of the study by Sampath Kuma et al. (SMD = -0.86) was significantly larger than that of the other three studies, which can be explained by five factors. (1) This study designated HOMA-IR as the primary outcome and calculated the sample size accordingly (>70 patients per group), providing adequate statistical power, whereas the other studies treated HOMA-IR as a secondary outcome with insufficient power (65). (2) The combined exercise performed 3–5 sessions per week can improve insulin sensitivity through dual mechanisms—increasing mitochondrial density and muscle mass (66). (3) The baseline HOMA-IR was 4.81, substantially higher than that in other studies, providing greater room for improvement (67). (4) The control group showed worsening of HOMA-IR from 4.63 to 5.38 (+16.2%), amplifying the relative effect of the exercise intervention. (5) The South Asian Indian population is known to have higher insulin resistance and lower muscle mass, potentially making them more responsive to exercise. It is important to note that a non-significant result should not be interpreted as evidence that “exercise has no effect on insulin resistance,” but rather as “the current evidence is insufficient to draw a definitive conclusion.” From a pathophysiological perspective, insulin resistance is a core feature of type 2 diabetes mellitus (68, 69), and the negative direction of the point estimate (SMD = -0.36) still suggests potential benefit. Furthermore, improving insulin sensitivity may require longer intervention periods (>12 weeks) or higher exercise doses than those used in the included studies (70, 71). Future large-sample, long-term randomized controlled trials are needed for further validation.

#### 4.1.4 Body mass index

After excluding the outlier study by Terauchi et al. (30) (Japan), exercise intervention significantly reduced BMI (SMD = -0.37, 95% CI: -0.61 to -0.12, P = 0.003, I² = 0%). Mean difference analysis showed that exercise intervention reduced BMI by approximately 0.99 kg/m² (95% CI: -1.64 to -0.34, P = 0.003) (**Supplementary Figure 2**). The study by Terauchi et al. (30) showed an effect direction opposite to that of other studies (SMD = +0.05), reporting a slight increase in BMI after exercise. This study was unusual in that the baseline BMI was only 26.7 kg/m² (overweight but not obese), whereas other studies included obese populations (BMI 30–34 kg/m²). The lower the baseline level, the smaller the room for BMI reduction (72). The magnitude of change in BMI in this study was very small (+0.13 kg/m²), likely representing measurement error or daily fluctuation rather than a true effect; the authors themselves noted that the weight change was not statistically significant. Moreover, an intervention duration of 12 weeks may be insufficient to induce significant BMI changes, particularly in non-obese populations (73). Comparison of intervention durations across studies revealed that Hordern et al. (48 weeks) showed the largest BMI reduction, whereas short-term studies of 6–12 weeks showed non-significant effects. Previous studies have also reported no significant improvement in BMI after 12 weeks of aerobic and resistance training (74) or 16 weeks of combined exercise (75). A meta-analysis by Boulé et al. similarly found no significant effect of exercise on BMI (76). In contrast, long-term interventions exceeding 6 months (e.g., the 48-week program by Hordern et al.) demonstrated beneficial effects on BMI (77). These findings suggest that sustained exercise for at least 6 months may be necessary to achieve measurable BMI improvements (73, 78).

#### 4.1.5 Maximal oxygen uptake (VO_2_max)

Exercise intervention significantly improved VO_2_max levels in patients with type 2 diabetes mellitus (SMD = 0.58, 95% CI: 0.34 to 0.82, P < 0.00001), with perfect consistency across studies (I² = 0%).Since its introduction by Hill in 1923, maximal oxygen uptake has been regarded as the international gold standard for assessing cardiorespiratory fitness (79, 80). An increase in VO_2_max reflects comprehensive improvements in cardiac output, oxygen delivery, and skeletal muscle oxidative capacity (81, 82). In patients with type 2 diabetes mellitus, VO_2_max is typically 15–20% lower than in healthy individuals, a finding that is associated with hyperglycemia-induced endothelial dysfunction, reduced mitochondrial density, and impaired oxygen utilization (83). Long-term regular aerobic training has been shown to increase VO_2_max in patients with type 2 diabetes mellitus (84). Boulé et al. reported that low-intensity aerobic exercise increased VO_2_max by more than 10% (76). Cauza et al. and Alam et al. also reported significant increases in VO_2_max following aerobic exercise and after six months of controlled exercise training, respectively (85, 86). In the present study, the 48-week combined aerobic and resistance exercise program by Hordern et al. (2009) contributed the highest weight (61.7%), further supporting the positive impact of long-term combined exercise on cardiorespiratory function. Low cardiorespiratory fitness is an independent predictor of all-cause mortality in patients with type 2 diabetes mellitus (87). Each 1 MET (3.5 mL/kg/min) increase in VO_2_max is associated with a 13–16% reduction in all-cause mortality risk (88) (89). The observed SMD of 0.58 corresponds to an approximate increase of 2–3 mL/kg/min in VO_2_max, which has clear prognostic implications.

### 4.2 Comparison with previous studies

The findings of this study are generally consistent with those of recently published meta-analyses. In a large-scale meta-analysis including 100 RCTs, also found that combined training was most effective in reducing HbA1c and that supervised exercise interventions were more effective than unsupervised programs (90). The present study further supports these conclusions and, through subgroup analysis, directly demonstrates that combined exercise is significantly superior to aerobic exercise alone (P for subgroup difference = 0.05). In a network meta-analysis comparing five exercise modalities on blood glucose and lipid outcomes, reported conclusions consistent with the present study (91). Compared with these studies, the unique contributions of the present study are: (1) directly comparing aerobic exercise with combined exercise, providing clearer clinical conclusions; (2) including body composition (BMI) and cardiorespiratory (VO_2_max) outcomes, offering more comprehensive evidence; (3) conducting in-depth analyses of heterogeneity sources and identifying specific outlier studies contributing to high heterogeneity; and (4) performing GRADE evidence grading for each outcome.

### 4.3 Limitations

First, heterogeneity was present in some analyses. Although we reduced most heterogeneity to acceptable levels through sensitivity and subgroup analyses, moderate-to-substantial heterogeneity remained in some analyses. This mainly stemmed from differences across studies in exercise type, intensity, frequency, intervention duration, and patient baseline characteristics. As can be clearly seen from the intervention characteristics of the included studies (**Tables 2**, **3**), the intervention periods ranged from 6 to 48 weeks, and the exercise frequency ranged from 2 days per week to 5–6 days per week. Although this diversity reflects real clinical practice, it also increased the heterogeneity of the pooled analyses.

Second, there is a lack of long-term follow-up data. All included randomized controlled trials reported outcome measures only immediately after the intervention period, without long-term follow-up data (e.g., ≥6 months post-intervention). This absence limits our ability to draw conclusions about the sustained benefits of exercise interventions. Therefore, the effect sizes presented in this meta-analysis should be interpreted as evidence of “short-term effectiveness” rather than “long-term sustainability.”

Third, the sample size for some outcome measures was small. The analysis of insulin resistance (HOMA-IR) was based on only four studies, resulting in insufficient statistical power. The pooled result did not reach statistical significance, which should not be interpreted as evidence that “exercise intervention has no effect on insulin resistance,” but rather as “the current evidence is insufficient to draw a definitive conclusion.”

Fourth, This systematic review was not prospectively registered in PROSPERO or other platforms, which is a limitation of this study. The main reason is that at the time of study initiation, we did not fully recognize the importance of prospective registration, and by the time we became aware of it, the literature search had already been completed. To mitigate this limitation, we strictly followed the PRISMA 2020 guidelines for reporting, with two reviewers independently completing the literature screening and data extraction, the analysis plan was predetermined before the analyses began, and all outcomes (including non-significant results) were fully reported. Nevertheless, the lack of prospective registration may still increase the risk of reporting bias. We recommend that future systematic reviews should be prospectively registered in PROSPERO or other platforms before study initiation to enhance research transparency and reproducibility.

Fifth, the number of included studies was limited for some outcomes. The limited number of studies for certain outcome measures precluded meaningful subgroup analyses or publication bias assessment (funnel plots require ≥10 studies).

Sixth, the geographic distribution of studies is unbalanced. Although this study included studies from multiple countries, most studies were from Asia (China, Japan, India, Iran, Pakistan, Sri Lanka) and Europe (the United Kingdom), lacking representation from North and South America. This may limit the generalizability of the findings to these regions.

### 4.4 Future directions

First, exercise intervention parameters should be reported in a standardized manner. Future studies should describe exercise protocols in detail, strictly adhering to the FITT principle (Frequency, Intensity, Time, Type). Crucially, exercise intensity should be quantified and monitored using objective measures, such as percentage of heart rate reserve (HRR) or percentage of maximal oxygen uptake (VO_2_max), rather than relying solely on subjective descriptions such as “moderate intensity” or “vigorous intensity.” From the included studies in this review, significant differences were observed in exercise type, duration, and frequency across studies, and this heterogeneity directly affects the interpretation of pooled results. Standardized and uniform reporting will help reduce between-study heterogeneity and facilitate result comparison and meta-analysis.

Second, long-term follow-up should be incorporated. All studies included in this meta-analysis reported outcome measures only immediately post-intervention, lacking long-term follow-up data (e.g., ≥6 months post-intervention). Only the studies by Hordern et al. (2009) (31) and Harihar et al. (2026) (41) had intervention periods longer than 12 weeks, but neither reported post-intervention follow-up data. Future studies should design observational follow-up periods of at least 6–12 months after the intervention to assess the long-term sustainability of exercise interventions.

Third, adequate power should be ensured for secondary outcomes. The current evidence for secondary outcomes such as insulin resistance (HOMA-IR) is insufficient (only 4 studies). Future studies should prespecify these measures as primary or key secondary outcomes and conduct adequate sample size calculations to provide robust evidence.

Fourth, multicenter, large-scale clinical trials are encouraged. Head-to-head multicenter randomized controlled trials comparing different standardized exercise protocols (e.g., high-intensity interval training vs. moderate-intensity continuous training) would provide the highest level of evidence for clinical guidelines. In this study, sample sizes varied considerably across studies, and large-sample studies had a substantial impact on the pooled results, suggesting that future studies should focus on rational sample size design.

Fifth, systematic reviews should be prospectively registered. Future systematic reviews and meta-analyses should be prospectively registered in platforms such as PROSPERO to enhance research transparency and reproducibility and to avoid duplicate publication.

Sixth, the geographic coverage of studies should be expanded. The studies included in this review were primarily from Asia, Africa, Europe, and Oceania, lacking representation from North and South America. Future studies should expand their geographic coverage to evaluate the consistency of the effects of exercise interventions across different ethnicities and healthcare systems.

### 4.5 Quality of the evidence

The Grading of Recommendations Assessment, Development and Evaluation (GRADE) system was applied to assess the quality of evidence for the following outcomes in this meta-analysis: glycated hemoglobin, fasting blood glucose, homeostatic model assessment for insulin resistance, body mass index, and maximal oxygen uptake. According to the GRADE assessment, the quality of evidence for HbA1c, FBG, VO_2_max, and BMI was rated as moderate, whereas the quality of evidence for HOMA-IR was rated as low (**Table 4**). The primary reasons for this assessment included the limited number of studies included for certain outcomes, the presence of moderate-to-substantial heterogeneity, and the fact that the confidence intervals for some outcomes included the null value. Specific factors contributing to this evaluation are detailed in **Table 4**.

**Table 4:** GRADE evidence quality assessment for each outcome.

Factor	HbA1c	FBG	HOMA-IR	BMI	VO2max
NO. of studies	8	7	3	4	4
Study design	RCT	RCT	RCT	RCT	RCT
Certainty assessment					
Risk of bias	Not serious	Not serious	Not serious	Not serious	Not serious
Inconsistency	Serious1	Not serious	Not serious	Not serious	Not serious
Indirectness	Not serious	Not serious	Not serious	Not serious	Not serious
Imprecision	Not serious	Not serious	Serious3	Not serious	Not serious
Publication bias	Undetected2	Undetected2	Undetected2	Undetected2	Undetected2
Other considerations	None	None	None	None	None
NO. of patients	627	767	320	269	293
Certainty	⨁⨁⨁◯	⨁⨁⨁◯	⨁⨁⨁◯	⨁⨁⨁◯	⨁⨁◯◯

Data are presented as number of studies or number of patients. ⨁⊕⊕⊕, high quality; ⨁⨁⨁◯, moderate quality; ⨁⨁◯◯, low quality; ⨁◯◯◯, very low quality. ¹Downgraded due to moderate heterogeneity. ²Downgraded due to serious imprecision. Publication bias was not formally assessed due to the limited number of included studies (<10 studies).

## 5 Conclusions

Exercise intervention significantly improved glycemic metabolic indices in T2DM patients, including lowering glycated hemoglobin (SMD=-0.46) and fasting glucose (SMD=-0.52), along with a small but significant reduction in BMI (SMD=-0.37) and a significant elevation in maximal oxygen uptake (SMD = 0.58). Although the improvement in HOMA-IR did not reach statistical significance (SMD=-0.18), it showed a trend toward improvement. Based on the American College of Sports Medicine (ACSM) guidelines and the evidence from this study, a comprehensive program of aerobic exercise (≥150 minutes of moderate intensity per week) combined with resistance training (2–3 times per week) is recommended for optimal results with sustained interventions for more than 12 weeks. In addition, the choice of exercise type should be based on individual treatment goals; a single form of exercise provides comparable improvement in glycemic control to combined training, but combined training is more advantageous in combining improvements in body composition and cardiorespiratory fitness. Limitations of this study include heterogeneity of exercise regimens among the included studies and lack of long-term follow-up data; future randomized controlled trials with standardized exercise parameters are needed and to explore the effects of different population characteristics on the effectiveness of exercise interventions. These findings provide an important evidence-based basis for the clinical development of personalized exercise prescription for T2DM, emphasizing that exercise interventions should be implemented with patient-specific conditions and long-term follow-up mechanisms should be established under the framework of ACSM guidelines.

## Data Availability

The original contributions presented in the study are included in the article/**Supplementary Material**. Further inquiries can be directed to the corresponding author.
