# Supplementary material for: Meta-analysis of the effects of exercise intervention on glucose metabolism and body composition in patients with type 2 diabetes mellitus
Source: Front Endocrinol (Lausanne). 2026 Jun 29;17:1835096. doi: 10.3389/fendo.2026.1835096 (PMC13357164; doi:10.3389/fendo.2026.1835096)
Supplement: Supplementary file 1 [file DataSheet1.docx]

**Supplementary materials**

Table 1 Change values from baseline of the included studies.

|  |  | Exercise Intervention | | | Control | | |
| --- | --- | --- | --- | --- | --- | --- | --- |
| HbA1c | Outcome measure | Exp Mean | Exp SD | Exp n | Cotr Mean | Cotr SD | Cotr n |
| Hordern 2014 | HbA1c | 0 | 0.7 | 88 | 0.7 | 1.7 | 88 |
| Mendham 2014 | HbA1c | 0.4 | 0.360555128 | 11 | 0.8 | 0.5 | 10 |
| Fayehun 2018 | HbA1c | 6.55 | -0.670625008 | 23 | 6.59 | 0.531875006 | 23 |
| Hu 2025 | HbA1c | -0.85 | 1.925798536 | 50 | -1.16 | 1.892960644 | 50 |
| Araiza 2006 | HbA1c | 0.2 | 1.769180601 | 15 | 0.1 | 1.571623365 | 15 |
| Amaravadi 2024 | HbA1c | -0.59 | 1.175542428 | 75 | 0.39 | 0.767398202 | 71 |
| Sinclair 2023 | HbA1c | -0.26 | 1.785525133 | 27 | -0.36 | 1.356355411 | 26 |
| Khanum 2019 | HbA1c | -0.2 | 1.233056365 | 32 | 0.23 | 1.415205639 | 32 |
| Lee 2015 | HbA1c | -0.345 | 1.1574 | 80 | 0.52 | 1.79016759 | 40 |
| RezkAllah 2019 | HbA1c | -1.07 | 0.4577 | 40 | 0.25 | 0.415812458 | 20 |
| Ranasinghe 2021 | HbA1c | -0.65 | 1.3762 | 56 | -0.5 | 1.4366628 | 30 |
| Anupama 2026 | HbA1c | -1.15 | 1.467376 | 42 | 0.67 | 1.64557 | 42 |
|  |  | Exercise Intervention | | | Control | | |
| FBS | Outcome measure | Exp Mean | Exp SD | Exp n | Cotr Mean | Cotr SD | Cotr n |
| Terauchi 2022 | FBG | -4.5 | 26.81175116 | 97 | 3.9 | 29.64101888 | 107 |
| Ezema 2014 | FBG | -31.97 | 31.38721874 | 30 | 2.92 | 34.85171588 | 24 |
| Amaravadi 2024 | FBG | -22.74 | 29.11950034 | 75 | -4.79 | 24.62041226 | 71 |
| Tapehsari 2020 | FBG | -15.92 | 17.2845943 | 47 | -0.21 | 27.19838966 | 48 |
| Khanum 2019 | FBG | -24.28 | 45.85389862 | 32 | -10.65 | 41.97869196 | 32 |
| Lee 2015 | FBG | -13.685 | 43.3727 | 80 | 6.5 | 61.64083144 | 40 |
| RezkAllah 2019 | FBG | -13.99 | 5.6135 | 40 | 2.62 | 6.245966699 | 20 |
| Ranasinghe 2021 | FBG | -8.2 | 44.6482 | 56 | -25.8 | 41.41014368 | 30 |
| Anupama 2026 | FBG | -30.4 | 74.8972 | 42 | 4.4 | 42.25719 | 42 |
|  |  | Exercise Intervention | | | Control | | |
| VO_2max_ | Outcome measure | Exp Mean | Exp SD | Exp n | Cotr Mean | Cotr SD | Cotr n |
| Hordern 2014 | VO_2max_ | 2.3 | 4.3 | 88 | 0.1 | 4.3 | 88 |
| Mendham 2014 | VO_2max_ | 3.7 | 8.252878286 | 11 | -0.5 | 5.850640991 | 10 |
| Ezema 2014 | VO_2max_ | 7.99 | 13.08037079 | 30 | 1.59 | 6.758461363 | 24 |
| Rezaeeshirazi 2021 | VO_2max_ | 4.7385 | 6.3561 | 27 | -0.52 | 3.429693864 | 15 |
|  |  | Exercise Intervention | | | Control | | |
| BMI | Outcome measure | Exp Mean | Exp SD | Exp n | Cotr Mean | Cotr SD | Cotr n |
| Terauchi 2022 | BMI | 0.29 | 12.74117734 | 97 | -0.3 | 12.41524466 | 108 |
| Hordern 2014 | BMI | -0.9 | 4.1 | 88 | 0.1 | 2.1 | 88 |
| Mendham 2014 | BMI | -4.5 | 3.05122926 | 11 | 0.2 | 6.702238432 | 10 |
| Araiza 2006 | BMI | -0.7 | 4.4 | 15 | -0.3 | 6.6 | 15 |
| Rezaeeshirazi 2021 | BMI | -0.8915 | 1.3903 | 27 | 0.0700000000000003 | 1.57467457 | 15 |
|  |  | Exercise Intervention | | | Control | | |
| Homa-IR | Outcome measure | Exp Mean | Exp SD | Exp n | Cotr Mean | Cotr SD | Cotr n |
| Terauchi 2022 | Homa-IR | -0.3 | 2.263249876 | 97 | 0.1 | 1.859758049 | 107 |
| Ranasinghe 2021 | Homa-IR | -0.03 | 1.032860107 | 28 | 0.26 | 1.040672859 | 30 |
| Ranasinghe 2021 | Homa-IR | 0.09 | 1.085725564 | 28 | 0.26 | 1.040672859 | 30 |
| Araiza 2006 | Homa-IR | -0.6 | 3.576310948 | 15 | -0.6 | 4.161730409 | 15 |
| Amaravadi 2024 | Homa-IR | -1.46 | 2.377540746 | 75 | 0.75 | 2.757299403 | 71 |
| Ranasinghe 2021 | Homa-IR | 0.03 | 1.0517 | 56 | 0.26 | 1.040672859 | 30 |
| Subgroup Study |  | Exercise Intervention | | | Control | | |
|  | Outcome measure | Exp Mean | Exp SD | Exp n | Cotr Mean | Cotr SD | Cotr n |
| Mix exercise |  |  |  |  |  |  |  |
| Amaravadi 2024 | HbA1c | -0.59 | 1.175542428 | 75 | 0.39 | 0.767398202 | 71 |
| Mendham 2014 | HbA1c | 0.4 | 0.360555128 | 11 | 0.8 | 0.5 | 10 |
| Ranasinghe 2021 | HbA1c | -8.2 | 44.6482 | 56 | -25.8 | 41.41014368 | 30 |
| Anupama 2026 | HbA1c | -30.4 | 74.8972 | 42 | 4.4 | 42.25719 | 42 |
| Hu 2025 | HbA1c | -0.85 | 1.925798536 | 50 | -1.16 | 1.892960644 | 50 |
| Hordern 2014 | HbA1c | 0 | 0.7 | 88 | 0.7 | 1.7 | 88 |
| Aerobic exercise |  |  |  |  |  |  |  |
| Lee 2015 | HbA1c | -0.345 | 1.1574 | 80 | 0.52 | 1.79016759 | 40 |
| RezkAllah 2019 | HbA1c | -1.07 | 0.4577 | 40 | 0.25 | 0.415812458 | 20 |
| Sinclair 2023 | HbA1c | -0.26 | 1.785525133 | 27 | -0.36 | 1.356355411 | 26 |
| Fayehun 2018 | HbA1c | 6.55 | -0.670625008 | 23 | 6.59 | 0.531875006 | 23 |
| Araiza 2006 | HbA1c | 0.2 | 1.769180601 | 15 | 0.1 | 1.571623365 | 15 |
| Khanum 2019 | HbA1c | -0.2 | 1.233056365 | 32 | 0.23 | 1.415205639 | 32 |

Table 2 Search strategy

| Database | Search strategy | Number |
| --- | --- | --- |
| PubMed | ((("Young Adult"[Mesh] OR "Adults, Young"[tiab] OR "Adult, Young"[tiab] OR "Young Adults"[tiab])) AND (("Diabetes Mellitus, Type 2"[Mesh] OR "Diabetes Mellitus, Type II"[tiab] OR "Type 2 Diabetes Mellitus"[tiab] OR "T2DM"[tiab]))) AND ((("Exercise"[Mesh] OR "Exercises"[tiab] OR "Exercise, Physical"[tiab] OR "Physical Exercise"[tiab] OR "Physical Exercises"[tiab] OR "Exercise, Aerobic"[tiab] OR "Aerobic Exercise"[tiab] OR "Aerobic Exercises"[tiab] OR "Exercises, Aerobic"[tiab] OR "Exercise, Isometric"[tiab] OR "Exercises, Isometric"[tiab] OR "Isometric Exercises"[tiab] OR "Isometric Exercise"[tiab] OR "Acute Exercise"[tiab] OR "Acute Exercises"[tiab] OR "Exercise, Acute"[tiab] OR "Exercises, Acute"[tiab] OR "Exercise Training"[tiab] OR "Exercise Trainings"[tiab] OR "Training, Exercise"[tiab] OR "Trainings, Exercise"[tiab] OR "Physical Activity"[tiab] OR "Activities, Physical"[tiab] OR "Activity, Physical"[tiab] OR "Physical Activities"[tiab]))) AND ("Randomized Controlled Trial"[Publication Type] OR "Randomized Controlled Trial"[tiab]) | 51 |
| Web Of Science | ((((((((((((((((((((((((TS=(Exercise)) OR TS=(Exercises)) OR TS=(Exercise, Physical)) OR TS=(Exercises, Physical)) OR TS=(Physical Exercise)) OR TS=(Physical Exercises)) OR TS=(Exercise, Aerobic)) OR TS=(Aerobic Exercise)) OR TS=(Aerobic Exercises)) OR TS=(Exercises, Aerobic)) OR TS=(Exercise, Isometric)) OR TS=(Exercises, Isometric)) OR TS=(Isometric Exercises)) OR TS=(Isometric Exercise)) OR TS=(Acute Exercise)) OR TS=(Acute Exercises)) OR TS=(Exercise, Acute)) OR TS=(Exercises, Acute)) OR TS=(Exercise Training)) OR TS=(Exercise Trainings)) OR TS=(Training, Exercise)) OR TS=(Trainings, Exercise)) OR TS=(Physical Activity)) OR TS=(Activities, Physical)) OR TS=(Activity, Physical)) OR TS=(Physical Activities)) AND (((TS=(Young Adult)) OR TS=(Adults, Young)) OR TS=(Adult, Young) OR TS=(Young Adults))) AND (((TS=(Diabetes Mellitus, Type 2)) OR TS=(Diabetes Mellitus, Type II)) OR TS=(Type 2 Diabetes Mellitus) OR TS=(T2DM))) AND TS=(Randomized Controlled Trial) | 395 |
| Embase | ('young adult'/exp OR 'adults, young':ti,ab,kw OR 'adult, young':ti,ab,kw OR 'young adults':ti,ab,kw) AND ('non insulin dependent diabetes mellitus'/exp OR 'diabetes mellitus, type ii':ti,ab,kw OR 'type 2 diabetes mellitus':ti,ab,kw OR 't2dm':ti,ab,kw) AND ('exercise'/exp OR 'exercises':ti,ab,kw OR 'exercise, physical':ti,ab,kw OR 'exercises, physical':ti,ab,kw OR 'physical exercise':ti,ab,kw OR 'physical exercises':ti,ab,kw OR 'exercise, aerobic':ti,ab,kw OR 'aerobic exercise':ti,ab,kw OR 'aerobic exercises':ti,ab,kw OR 'exercises, aerobic':ti,ab,kw OR 'exercise, isometric':ti,ab,kw OR 'exercises, isometric':ti,ab,kw OR 'isometric exercises':ti,ab,kw OR 'isometric exercise':ti,ab,kw OR 'acute exercise':ti,ab,kw OR 'acute exercises':ti,ab,kw OR 'exercise, acute':ti,ab,kw OR 'exercises, acute':ti,ab,kw OR 'exercise training':ti,ab,kw OR 'exercise trainings':ti,ab,kw OR 'training, exercise':ti,ab,kw OR 'trainings, exercise':ti,ab,kw OR 'physical activity':ti,ab,kw OR 'activities, physical':ti,ab,kw OR 'activity, physical':ti,ab,kw OR 'physical activities':ti,ab,kw) AND ('randomized controlled trial'/exp OR 'randomized controlled trial':ti,ab,kw) | 38 |
| Cochrane Library | MeSH descriptor: [Young Adult] explode all trees OR (Adults, Young):ti,ab,kw or (Adult, Young):ti,ab,kw or (Young Adults):ti,ab,kw AND MeSH descriptor: [Diabetes Mellitus, Type 2] explode all trees OR (Diabetes Mellitus, Type II):ti,ab,kw or (Type 2 Diabetes Mellitus):ti,ab,kw or (T2DM):ti,ab,kw AND MeSH descriptor: [Exercise] explode all trees OR (Exercises):ti,ab,kw or (Exercise, Physical):ti,ab,kw or (Exercises, Physical):ti,ab,kw or (Physical Exercise):ti,ab,kw or (Physical Exercises):ti,ab,kw or (Exercise, Aerobic):ti,ab,kw or (Aerobic Exercise):ti,ab,kw or (Aerobic Exercises):ti,ab,kw or (Exercises, Aerobic):ti,ab,kw or (Exercise, Isometric):ti,ab,kw or (Exercises, Isometric):ti,ab,kw or (Isometric Exercises):ti,ab,kw or (Isometric Exercise):ti,ab,kw or (Acute Exercise):ti,ab,kw or (Acute Exercises):ti,ab,kw or (Exercise, Acute):ti,ab,kw or (Exercises, Acute):ti,ab,kw or (Exercise Training):ti,ab,kw or (Exercise Trainings):ti,ab,kw or (Training, Exercise):ti,ab,kw or (Trainings, Exercise):ti,ab,kw or (Physical Activity):ti,ab,kw or (Activities, Physical):ti,ab,kw or (Activity, Physical):ti,ab,kw or (Physical Activities):ti,ab,kw AND (Randomized Controlled Trial):ti,ab,kw | 254 |


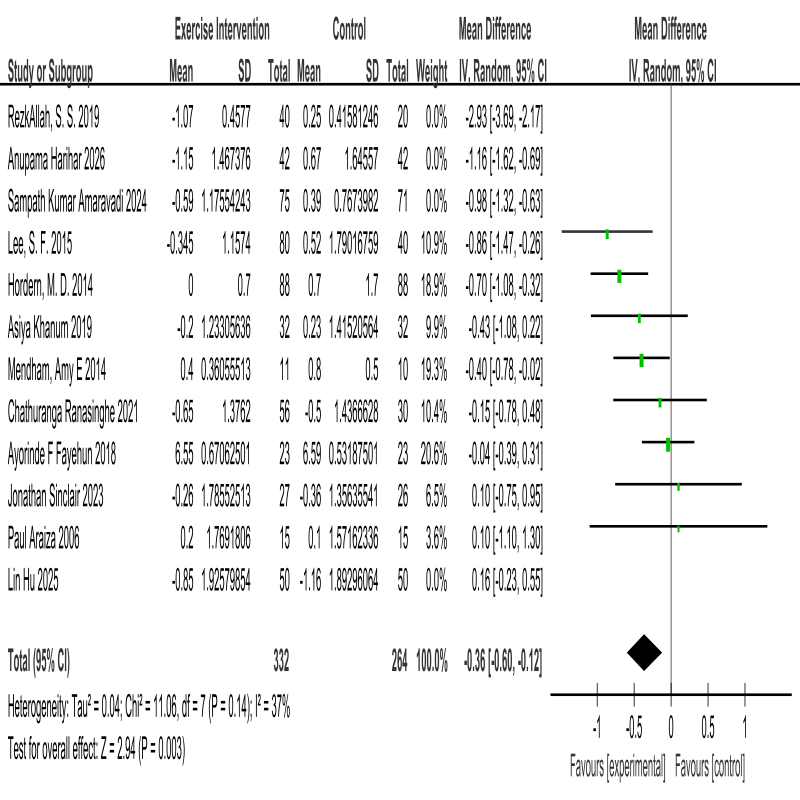


Figure 1 Forest plot of mean difference (MD) for glycated hemoglobin (HbA1c) using a random-effects model.


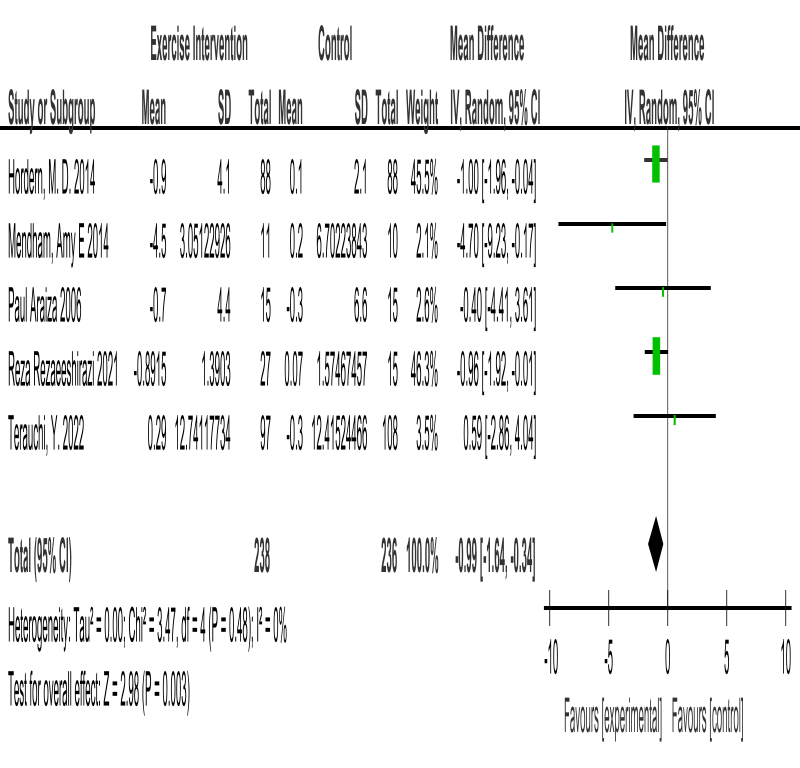


Figure 2 Forest plot of mean difference (MD) for body mass index (BMI) using a random-effects model.
